# Improvement of Metabolic-Associated Fatty Liver Disease by Magnetic Resonance Spectroscopy in Morbidly Obese Women Undergoing Roux-en-Y Gastric Bypass, following a Postoperative Mediterranean-like Diet

**DOI:** 10.3390/nu16142280

**Published:** 2024-07-16

**Authors:** Jaime Ruiz-Tovar, Carolina Llavero, Maria Rodriguez-Ortega, Nuria M. De Castro, Maria Cristina Martín-Crespo, Gema Escobar-Aguilar, Ana Martin-Nieto, Gilberto Gonzalez

**Affiliations:** 1 San Juan de Dios Foundation, 28036 Madrid, Spain; mrortega@comillas.edu (M.R.-O.); ndecastro@comillas.edu (N.M.D.C.); mmartinc@comillas.edu (M.C.M.-C.); gemaescobar@comillas.edu (G.E.-A.); amartinn@comillas.edu (A.M.-N.); 2 Health Sciences Department, San Juan de Dios School of Nursing and Physical Therapy, Comillas Pontifical University, 28036 Madrid, Spain; 3 Obesity Unit, Garcilaso Clinic, 28010 Madrid, Spain; carolinallavero@gmail.com; 4 Hospital Real San José, Guadalajara 19001, Mexico; gilpchmd@yahoo.com.mx

**Keywords:** liver steatosis, metabolic-associated fatty liver disease, MAFLD, magnetic resonance spect5roscopy, Roux-en-Y gastric bypass, Mediterranean diet, adherence, morbid obesity, bariatric surgery

## Abstract

(1) Background: Bariatric surgery has demonstrated the capacity to improve metabolic-associated fatty liver disease (MAFLD) in patients with morbid obesity. In addition, the Mediterranean diet contains anti-inflammatory, anti-oxidative, and anti-fibrotic components, promoting a beneficial effect on MAFLD. This study aimed to assess the improvement of MAFLD, specifically liver steatosis, in morbidly obese patients undergoing Roux-en-Y gastric bypass (RYGB) and following a hypocaloric Mediterranean-like diet. (2) Methods: A prospective observational pilot study of 20 patients undergoing RYGB was conducted. The participants underwent a magnetic resonance spectroscopy study 2 weeks before the surgical act and one year postoperatively to assess the percentage of lipid content (PLC). The adherence to the Mediterranean diet was determined by the KIDMED test 1 year after surgery. (3) Results: Mean baseline PLC was 14.2 ± 9.4%, and one year after surgery, it decreased to 4.0 ± 1.8% (*p* < 0.001). A total of 12 patients (60%) were within the range of moderate adherence to the Mediterranean diet, whereas 8 patients (40%) showed a high adherence. The patients with high adherence to the Mediterranean diet presented significantly lower values of postoperative PLC. (4) Conclusions: Liver steatosis significantly reduces after RYGB. This reduction is further improved when associated with a high adherence to a Mediterranean diet.

## 1. Introduction

Metabolic-associated fatty liver disease (MAFLD) consists in the deposition of lipids inside the hepatocytes, which exceeds 5% of the total liver weight. The term MAFLD includes different entities, including the first phase of simple steatosis, which is a relatively benign condition. The deposition of lipids can be identified by the immune system as a foreign body and an inflammatory response starts, leading to the phase of non-alcoholic steatohepatitis (NASH). A chronic inflammatory process evolves into pericellular or perisinusoidal fibrosis, as during the repair, the damaged hepatocytes are replaced by fibrotic tissue. In some cases, these lesions might progress to liver cirrhosis, similar to that of alcoholic hepatitis. The mechanism of MAFLD production still remains unclear, but it has been associated with insulin resistance, the release of inflammatory cytokines, and the development of an oxidative stress environment [1,2].

MAFLD is closely related to obesity and is actually the most common liver disorder in developed countries [3]. As a result of the current obesity epidemic, hepatic steatosis has become a common health problem, affecting all age groups, from children to the elderly. The prevalence of MAFLD in developed countries is estimated to affect approximately 25–30% of the adult population. This percentage can increase significantly in at-risk populations, such as people with obesity or diabetes, reaching up to 60–80%. [4].

MAFLD has been shown to lead to frequent alterations in lipid metabolism and increased cardiovascular risk with the acceleration of atherosclerosis and related events. MAFLD is associated with insulin resistance, metabolic syndrome and its components, as well as proatherogenic lipid alterations. It is well established that lipid ratios, total cholesterol/HDL-cholesterol, and triglycerides/HDL-cholesterol, are strong predictors of cardiovascular risk (CVR). Using these indices, it has been determined not only that cardiovascular risk is increased in patients with MAFLD but that it progresses in parallel with progression to steatohepatitis. Moreover, MAFLD results in an early increase in carotid intima-medial thickness and a higher prevalence of atheromatous plaques. It has been shown that patients with MAFLD also suffer from endothelial dysfunction, which is not dependent on obesity or the presence of metabolic syndrome or any of its components [5,6].

Bariatric surgery has been shown to achieve complete remission or at least significant improvement of MAFLD in a high percentage of morbidly obese patients, mostly related to weight loss and the reduction in adiposity [5]. The procedure of choice for assessing MAFLD is a histopathological examination of a liver biopsy and a semi-quantitative estimation of the percentage of hepatocytes containing fatty macrovesicles. However, obtaining an intraoperative biopsy during bariatric surgery carries inherent risks. Furthermore, performing a postoperative percutaneous liver biopsy to assess potential improvement is an invasive procedure that the patient may not consent to [7].

Non-invasive alternatives for diagnosing and monitoring hepatic steatosis include biochemical markers of hepatic steatosis [8] or imaging techniques such as ultrasound, computed tomography scan (CT scan), or magnetic resonance imaging (MRI). MRI-based techniques, including magnetic resonance spectroscopy (MRS), appear to be superior to other imaging techniques. MRS allows for accurate quantification of fat content in the liver, even at low levels of steatosis (from 3% fat content and above), with higher sensitivity and specificity for detecting and quantifying liver fat, outperforming other techniques such as ultrasound and CT. In addition to fat quantification, MRS can provide information on other liver characteristics, such as fibrosis and inflammation. Actually, there is increasing evidence that the MRS results, in terms of an intrahepatocitary lipid content assessment, are comparably more valuable than a liver biopsy but with the advantage that MRS is a non-invasive and safe technique. However, MRS also has certain disadvantages, such as higher cost, lower accessibility due to lack of equipment in all hospitals, longer examination time, claustrophobia that some patients may experience, and limitations in morbidly obese patients. [9,10].

It is widely known that good adherence to the Mediterranean diet is associated with higher levels of physical and psychological health, greater longevity, and less development of obesity [11]. A review paper, evaluating different types of diets, placed the Mediterranean diet as number one in the ranking of model diets for reducing cardiovascular risk [12]. The traditional Mediterranean diet is characterized by a high intake of fruits, vegetables, legumes, nuts and cereals, a moderate ingestion of fish, and a low intake of meat and sweets, where olive oil is the main source of lipids in the diet [13]. Many of these food items contain a diverse array of phytonutrients, with polyphenols and vitamins standing out as particularly significant contributors. The traditional Mediterranean diet is abundant in antioxidant compounds such as vitamin E, β-carotene, vitamin C, and flavonoids, as well as minerals like selenium and natural folate [14]. Furthermore, the Mediterranean diet is regarded as environmentally sustainable [15].

Unfortunately, even in Mediterranean countries, adherence to the Mediterranean diet is decreasing. The modernization of society has led to a series of sociological and cultural changes that inevitably affect eating habits and preferences. Less time is devoted to food shopping and meal preparation, and instead, processed foods are preferred, which generally involve excessive consumption of animal-derived foods, especially meats and their products and refined sugars, resulting in an increase in saturated fats and cholesterol in the diet [16]. This, added to an increasingly sedentary lifestyle, has led to an increase in the prevalence of diabetes, being overweight, and even morbid obesity [17].

As previously mentioned, weight loss is the most effective method of reducing the fat content of the liver. Accordingly, the weight loss induced by bariatric surgery is probably the main reason for the improvement or remission of MAFLD. A previous study demonstrated that a high adherence to a Mediterranean-like diet in the postoperative course of sleeve gastrectomy, as a bariatric procedure, was associated with greater weight loss and improved lipid profile [18]. Furthermore, the improvement in the serum lipid profile, with a reduction in total cholesterol and LDL-cholesterol, and an increase in HDL-cholesterol, prevents the deposition of fatty liver from an imbalance between lipid accumulation and elimination, driven by hepatic triglyceride synthesis and de novo lipogenesis. The Mediterranean diet contains anti-inflammatory, anti-oxidative, and anti-fibrotic components, promoting a beneficial effect in managing MAFLD [19,20]. Although there are not a large number of papers published in the literature analyzing the impact of the Mediterranean diet on patients undergoing bariatric surgery, a recent narrative review studied the effect of adherence to the Mediterranean diet both before and after bariatric surgery, concluding that not only is the diet adequate and safe but that the greater the adherence, the greater the weight loss, both preoperatively and postoperatively. Consequently, MAFLD may improve further with a higher adherence to a Mediterranean diet [21].

This study aimed to assess the improvement of MAFLD, and specifically liver steatosis, in patients with morbid obesity undergoing Roux-en-Y gastric bypass, according to the degree of adherence to a Mediterranean-like diet.

## 2. Materials and Methods

A prospective observational pilot study of 20 patients undergoing laparoscopic Roux-en-Y gastric bypass as a bariatric technique was conducted.

Inclusion criteria were females, with a body mass index (BMI) between 35 kg/m^2^ and 45 kg/m^2^, and an ultrasound diagnosis of hepatic steatosis. Exclusion criteria included an alcohol intake of more than 20 g per day, intake of hepatotoxic substances, chronic hepatitis derived from virus infection or other causes, or any other known liver disorders, carrying a pacemaker, an intracorporeal electronic device, or any metallic implant not compatible with MRI. Claustrophobia was also considered an exclusion criterion, as well as patients with a BMI ≥ 45 kg/m^2^ in the event of difficulty entering the MRI machine due to their body volume.

The sample size was limited to 20 patients due to the financial constraints of the project to be able to perform MRS. Nevertheless, a sample size calculation was performed to confirm that the inclusion of 20 patients was sufficient to confirm a significant decrease in hepatic steatosis. This was based on our group’s previous study on the reduction in hepatic steatosis after sleeve gastrectomy, in which the preoperative prevalence of steatosis was 64% and was reduced to only 8% [10]. Assuming similar data after RYGB, with a statistical power of 80% and a significance *p* < 0.05, it was necessary to include 10 patients in each group. Therefore, the sample size of 20 patients seemed sufficient.

### 2.1. Surgical Technique

A laparoscopic approach was performed on all the patients. A 6 cm long gastric pouch was made and calibrated with a 36 Fr bougie. A 60 cm long biliary limb and a 150 cm long alimentary limb were made, calibrating the gastrojejunal anastomosis to 2 cm. The Roux-en-Y gastric bypass achieves its weight loss effect through a combination of a restrictive effect, which limits the amount of food that can be eaten, and a malabsorptive effect, in which part of the small intestine is rerouted so that food passes directly from the small stomach pouch into the jejunum, bypassing the duodenum and part of the small intestine. This reduces the absorption of calories and nutrients. In addition, these anatomical changes alter intestinal hormones, which help to reduce appetite, improve satiety, and improve glucose regulation. The improvement in hepatic steatosis after RYGB is due in part to significant weight loss, as the reduction in body weight reduces the amount of fat accumulated in the liver, improvement in insulin resistance, which reduces fat accumulation in the liver, hormonal changes due to the modification of the digestive tract affecting hormones that regulate glucose and lipid metabolism, and reduction in inflammation [5].

### 2.2. Magnetic Resonance Spectroscopy

All participants underwent magnetic resonance spectroscopy (MRS) examinations two weeks before the intervention and 12 months postoperatively. The examinations were conducted using a Philips Intera 1.5 T device equipped with a standard SENSE body coil. MRE was performed using the single voxel technique, with two voxels positioned in different regions of the left and right hepatic lobes to ensure a more comprehensive and uniform distribution of intrahepatic fat. The average voxel volume was 30 × 30 × 30 mm and 20 × 20 × 20 mm for voxels placed in the right and left lobes, respectively. After automatic selective volume clipping, spectra were obtained using the point-resolved spectroscopy technique (PRESS) with parameters set to TR/TE/scans = 2000 ms/35 ms/64, without water suppression. Each spectrum comprised 1024 complex data points in the time domain with a spectral width of 2000 Hertz.

Spectral analysis was performed offline in the time domain using the MRUI 7.0 software package [9,10]. The diagnosis of steatosis by MRS was determined based on specific criteria: no steatosis when the percentage of lipid content (PLC) was less than 5%, mild steatosis when the PLC ranged from 5% to 10%, moderate steatosis when the PLC fell within the range of 10% to 30%, and severe steatosis when the PLC exceeded 30%, as previously outlined in the literature [9,10].

### 2.3. Mediterranean Diet

A liquid diet was prescribed for the first 2 weeks postoperatively. On the 3rd and 4th week, a semi-solid diet was progressed to include soft, easily chewed foods. From one month after surgery, a regular Mediterranean pattern diet was prescribed to all the patients with calorie adjustment according to basal metabolic expenditure, calculated using the Harris–Benedict formula, and with a daily restriction of 300 Kcal on this basal metabolic expenditure. The prescribed diet followed a balanced Mediterranean-style pattern (its percentage composition consisted of 51% carbohydrates, 23% protein, and 26% fat) with a high intake of fruit and vegetables and a low intake of meat and oil, with olive oil being the main source of fat, as previously described [22]. The percentage of monounsaturated fatty acids delivered with the diet was 17%, saturated fatty acids 5%, and polyunsaturated fatty acids 4%. The main difference between the Mediterranean-like diet prescribed to our patients and a traditional Mediterranean diet is the absence of a glass of wine and the limitation or even elimination of nuts, so as not to exceed the caloric value. The list of foods included in the prescribed diet is shown in Table 1. No diets other than the Mediterranean-style diet were prescribed.

Patients followed a regimen of visits with the nutritionist at 1, 2, 3, 6, 9, and 12 months after surgery for the first year. During these visits, questions were answered, dietary compliance was analyzed, and nutritional advice was given.

### 2.4. Assessment of Adherence to the Mediterranean Diet

All patients completed the KIDMED test 1 year after surgery before undergoing the postoperative MRS. The KIDMED test assesses adherence to the Mediterranean diet, analyzing the Mediterranean dietary patterns (daily consumption of fruit and vegetables, weekly intake of fish and legumes) and patterns contrary to the Mediterranean diet (frequent consumption of ready-made, precooked food and sweets).

The test includes 16 questions to be answered YES or NO. Questions with a negative connotation were assigned a value of −1, and those with a positive connotation were assigned a value of +1. A total test score between 0–3 reflects poor adherence, between 4–7 moderate, and between 8–12 good adherence to the principles of the Mediterranean diet [23].

### 2.5. Variables

Preoperative age and comorbidities were recorded. Anthropometric and PLC parameters, determined by MRS, were collected preoperatively and 1 year after surgery. Anthropometric measurements included preoperative weight and BMI and weight loss, BMI and excess weight loss (EWL) at 1 year after surgery. In addition, biochemical parameters, including aspartate aminotransferase (AST), alanine aminotransferase (ALT), serum cholesterol, triglyceride, HDL-cholesterol, LDL-cholesterol, fasting glucose, and glycated hemoglobin, were analyzed at baseline and 1 year after surgery.

### 2.6. Statistical Analysis

All statistical analyses were performed with SPSS version 28.0 (SPSS Inc., Chicago, IL, USA). Results were presented as mean ± standard deviation or number and percentages in non-Gaussian variables. The Gaussian distribution was tested using the Kolmogorov–Smirnov test. A paired Student’s *t*-test and Friedman’s test were used to compare quantitative variables before and after surgery. Student’s *t*-test, for independent variables, and the Mann–Whitney test were used to compare data between the groups with different adhesion to the Mediterranean diet. A *p*-value < 0.05 was considered statistically significant.

The local Ethics Committee approved the study, and all the patients signed a written informed consent before entering the study.

## 3. Results

A total of 20 females were included in the study with a mean age of 42.7 ± 10.3 years. Preoperative comorbidities included hypertension (30%), type 2 diabetes mellitus (30%), dyslipidemia (50%), osteoarthritis (20%), and sleep apnea—hypopnea syndrome (SAHS) (15%).

### 3.1. Adherence to the Mediterranean Diet

**Figure 1:**
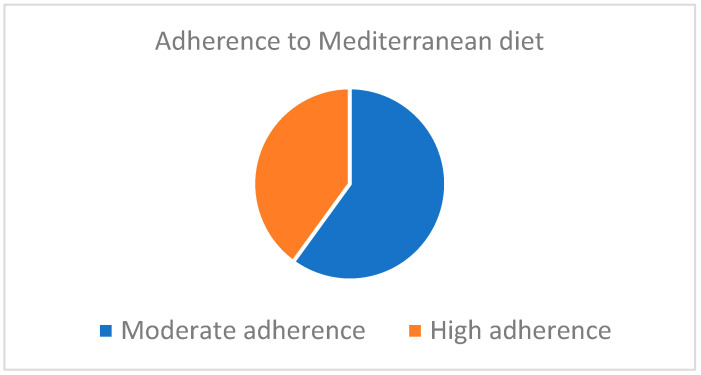
Adherence to the Mediterranean diet according to the KIDMED test.

According to the postoperative score obtained in the KIDMED test, 12 patients (60%) were within the range of moderate adherence to the Mediterranean diet, whereas 8 patients (40%) showed a high adherence (Figure 1).

Evaluating independently the different items analyzed in the KIDMED test, we found it striking that, overall, the worst adherence is to the intake of pasta, rice, and dairy products, as these foods are usually less well tolerated after bariatric surgery. However, the main differences between the groups with moderate and high adherence to the Mediterranean diet, although without reaching statistical significance due to insufficient sample size, is that the group with high adherence consumes more fruit, vegetables, pulses, and fish, while in the group with moderate adherence, there is some intake of fast food, sweets, or candies (Table 2).

### 3.2. Anthropometric Measurements

The mean preoperative weight was 107.3 ± 18.3 Kg, and the mean preoperative BMI was 41.4 ± 3.4 Kg/m^2^. One year after surgery, the mean weight was 75.1 ± 9.4 Kg, and the mean BMI was 29.0 ± 2.6 Kg/m^2^, with a mean excess weight loss of 32.2 ± 7.3 Kg and a mean percentage of excess weight loss of 75.7 ± 5.2%. The distribution of anthropometric values according to the adherence to the Mediterranean diet is shown in Table 3.

### 3.3. MRS Measurements

Mean preoperative PLC was 14.3 ± 9.3%, and 1 year after surgery, these values decreased to 4.0 ± 1.8% (*p* < 0.001). Although all the patients presented a preoperative ultrasonographic diagnosis of liver steatosis, according to the previously mentioned classification of steatosis depending on the percentage of intrahepatocitary lipid content, only 16 patients (80%) showed preoperative PLC values in a range of steatosis (10 patients were classified as with mild steatosis and 6 with moderate steatosis). One year after surgery, PLC values from all the patients were within the normal range, except in one case with preoperative moderate steatosis, presenting mild steatosis postoperatively. 

Despite this, in both groups, a significant reduction in PLC was observed, and significantly lower values were obtained in the patients with high adherence to the Mediterranean diet (Table 4).

### 3.4. Biochemical Parameters

At baseline, increased AST levels (>40 U/I) were found in 15% of the patients, increased ALT (>40 U/I) in 30%, increased triglyceride (>150 mg/dL) in 20%, and decreased HDL-cholesterol (<40 mg/dL) in 35%. Postoperatively, only one patient (5%) remained with elevated AST and ALT levels. The values of the glycemic and lipid profile remained within the normal range postoperatively. It is noteworthy that all the patients with a preoperative diagnosis of type 2 diabetes or dyslipidemia were under pharmacological treatment, while all the postoperative determinations were in the absence of any treatment (Table 5).

According to the stratification of the sample in the categories of moderate and high adherence to the Mediterranean diet, there were no significant differences in the preoperative values of any of the biochemical parameters analyzed. However, in the postoperative values, significantly lower levels of both liver enzymes, triglycerides, and fasting glucose were observed in the group with a high adherence to the Mediterranean diet. In addition, there was a trend towards significantly higher levels of HDL-cholesterol in the high adherence group (Table 6).

## 4. Discussion

In the etiopathogenesis of MAFLD, the liver plays a central role in lipid metabolism, capturing free fatty acids from plasma, which, if not utilized as an energy source by oxidation, are stored or exported after lipid and lipoprotein synthesis. A series of alterations of local and systemic factors, which control the balance between lipid influx, oxidation, and export, lead to hepatic accumulation of triglycerides.

Insulin resistance and obesity, primarily of the visceral type, are two important elements involved in the development of MAFLD. Both increase the influx of free fatty acids into the liver, leading to increased hepatic triglyceride production. Moreover, hyperinsulinemia and hyperglycemia, which often accompany insulin resistance, can also promote de novo free fatty acid lipogenesis by overexpressing lipogenic transcription factors such as sterol regulatory element-binding protein (SREBP-1c) or carbohydrate response element-binding protein [24]. Free fatty acids (FFA) that are not incorporated into triglycerides must be metabolized by oxidation in mitochondria, peroxisomes, and microsomes. However, activation of SREBP-1c increases malonyl-Coenzyme A, which inhibits free fatty acid oxidation. The net result of these three alterations is the increased hepatic availability of free fatty acids as a substrate for triglyceride synthesis [25]. In this synthesis, the enzyme acyl-Coenzyme A:diacylglycerol acyltransferase (DGAT) plays a key role by catalyzing the final step toward the esterification of free fatty acids to triglycerides. DGAT consists of two primary isoforms (DGAT1 and DGAT2). While DGAT1 is present in several tissues, DGAT2 is specific to hepatocytes. Experiments in mice have shown that its overexpression causes steatosis, while its inhibition reverses steatosis. After esterification, free fatty acids are finally packaged and stored as triglyceride vacuoles in hepatocytes, or after binding to apolipoprotein B, mediated by microsomal triglyceride transfer protein (MTTP), they are released into the bloodstream as very low-density lipoproteins (VLDL). Since SREBP-1c inhibits MTTP formation, insulin resistance by overexpressing SREBP-1c contributes to lower apolipoprotein B incorporation and consequent lower VLDL formation. There may also be a decrease in apolipoprotein B synthesis or a reduced postprandial apolipoprotein B response dissociated from the increase in triglycerides. In all cases, the ultimate consequence is difficulty in the delivery of excess triglycerides into the plasma. When the rate of triglyceride synthesis exceeds the capacity for VLDL production and export, triglycerides accumulate within hepatocytes, leading to steatosis [5,26].

Derived from the presence of insulin resistance in MAFLD, overproduction of VLDL results in a serum lipid profile of fatty liver subjects characterized by elevated triglyceride levels, low HDL cholesterol, and an increase in small, dense LDL particles. Hypertriglyceridemia is the most common laboratory abnormality. In the general population, the finding of hypertriglyceridemia or mixed dyslipidemia increases the likelihood of fatty infiltration on liver ultrasound by a factor of 5.9 and 5.1, respectively. Although MAFLD is associated with obesity, preferably visceral, the characteristic lipid findings are independent of obesity. The presence of hepatic steatosis in patients with type 2 diabetes mellitus aggravates diabetic dyslipidemia, independently of hyperglycemia [26].

The improvement of hepatic steatosis following bariatric surgery has been widely demonstrated. Despite most evidence being based on Roux-en-Y gastric bypass and malabsorptive procedures, improvement has also been reported after restrictive procedures. In a previous study by our group, analyzing the evolution of hepatic steatosis after sleeve gastrectomy, we observed that the mean preoperative PLC was 14.2%, and 6 months after surgery, these values decreased to 4.3%. These results are similar to those obtained in the present study. It is true that both studies are not equivalent given that, in the previous study, the evaluation of PLC was performed 6 months after surgery, while in the current study, it was performed 12 months after surgery. However, given that during the first 6 months after surgery, an average loss of 66% of excess weight is achieved, and that the reduction in steatosis is closely associated with weight loss, we would not expect very different results at 12 months after sleeve gastrectomy [10].

While significant weight loss appears to be the primary driver behind the reduction in intrahepatocellular lipid content, the improvement of steatosis can also be greatly influenced by enhancements in the lipid profile resulting from duodenal exclusion or small bowel bypassing [27,28]. There is ample evidence in the literature supporting the idea that RYGB improves type 2 diabetes mellitus through mechanisms beyond weight loss [29,30,31]. This is evidenced by the fact that duodenal exclusion induces substantial changes in gut hormones, bile acids metabolism, and gut microbiota [32,33]. These changes collectively contribute to the regulation of insulin sensitivity, insulin secretion, and appetite control.

In addition to its effects on type 2 diabetes mellitus, the exclusion of the proximal small intestine has been shown to reduce liver fat accumulation by downregulating various mechanisms involved in lipid deposition in both the liver and skeletal muscle. Importantly, these effects are not solely mediated by weight loss or reduced calorie intake. Abnormal accumulation of lipids in the liver is a key characteristic of MAFLD progression [34]. These lipids are stored in cytoplasmic lipid droplets, with perilipin 2 (PLIN2) serving as the major coat protein [33]. PLIN2 plays a crucial role in preventing lipid breakdown by lipases and is highly expressed in the livers of MAFLD patients [35].

Furthermore, the interaction of PLIN2 with lysosomal membrane-associated protein 2 (LAMP2A) is essential for PLIN2 catabolism, leading to the exposure of lipid droplets to lipases and thereby influencing lipid storage and metabolism [33]. Bariatric procedures involving duodenal exclusion, such as duodenojejunal bypass, have been associated with increased LAMP2A protein expression and reduced PLIN2 expression, resulting in decreased liver fat deposition and intrahepatocellular triglyceride content. Moreover, similar protein expression patterns have been observed in skeletal muscle, indicating a potential role of PLIN2 in regulating glucose uptake and insulin resistance. Overall, these findings suggest that PLIN2 may play a pivotal role in modulating both liver fat deposition and insulin resistance, two interconnected features of MAFLD progression [36].

As previously mentioned, the routine assessment of MAFLD is performed using biochemical parameters or imaging tests. Among the latter, ultrasonography is the most frequently conducted, as it is easy to perform, cheap, non-invasive, and available at most medical institutions. However, our results demonstrate that ultrasonographic diagnosis accuracy is insufficient. All the patients included in our study had a preoperative ultrasonographic diagnosis of liver steatosis. However, MRS has shown that liver steatosis was overestimated in our patients, as 20% of them presented preoperative PLC within the normal range. This observation has been previously reported in other studies [10]. In our opinion, ultrasonography is not the most appropriate approach to obtain a more accurate diagnosis of liver steatosis. As it tends to overestimate the liver affection, in such cases with the ultrasonographic suspicion of hepatic steatosis, MRS is recommended to confirm the diagnosis, without the need for invasive procedures such as liver biopsy.

Several biological mechanisms can be proposed to explain why a high adherence to the Mediterranean diet is associated with a greater reduction in hepatic steatosis. The accumulation of lipids in the hepatocytes leads to an inflammatory response that, maintained over time, leads to NASH. Foods typical of the Mediterranean diet, such as fruits, vegetables, nuts, and fish, contain antioxidants and anti-inflammatory compounds that can reduce liver and systemic inflammation [19,20].

The Mediterranean diet, rich in monounsaturated and polyunsaturated fats (such as olive oil and omega-3 fatty acids), improves insulin sensitivity, which helps to reduce fat accumulation in the liver, even without weight loss [37]. Diverse components of the Mediterranean diet have shown a clear benefit in controlling the pathophysiological mechanisms that lead to MAFLD [38]. Some of the main components of the Mediterranean diet with proven metabolic effects are whole grains with a low glycemic index and foods rich in unsaturated fatty acids and their content of phytochemical compounds. Wholegrain cereals, high in dietary fiber, interact with carbohydrate metabolism and the associated insulin response. Several clinical trials on diabetes have shown the beneficial effect that oleuropein or olive leaf extracts may have against type 2 diabetes mellitus. These studies reported a significant reduction in blood glucose levels and glycated hemoglobin. Additionally, oleuropein can promote glucose-stimulated insulin secretion in pancreatic β cells.

As a balanced and nutrient-rich diet, it facilitates weight loss and maintenance of a healthy weight, which is crucial for reducing hepatic steatosis. Dietary fiber regulates the absorption of cholesterol and glucose and contributes to satiety to facilitate weight control. In addition, this diet helps to improve blood cholesterol and triglyceride levels, which reduces the amount of fat deposited in the liver. Unsaturated fatty acids improve lipid metabolism at the liver level, while phytochemical compounds, such as dietary polyphenols, have shown some anti-inflammatory effects. Inadequate dietary intake of antioxidants may heighten the risk of atherosclerotic plaque formation due to alterations in lipoprotein oxidation [39,40,41]. Lycopene (carotenoid) from fruits, vegetables, legumes, and whole grains has been shown to possess antioxidant activity in hepatocytes, reducing lipid peroxidation and inflammation and improving insulin sensitivity [42]. Finally, the Mediterranean diet promotes a healthy gut microbiota, which can positively influence liver metabolism and reduce fat accumulation in the liver.

In a review study on the effect of the Mediterranean diet on histological indicators and imaging tests in non-alcoholic fatty liver disease, they found that the Mediterranean diet was associated with a decrease in the percentage of intrahepatic lipids and a lower degree of liver damage, determined by biopsy, in proportion to the patient’s adherence to the Mediterranean diet. Studies evaluating the effect of diet on hepatic steatosis by magnetic resonance imaging showed a reduction in liver fat content in subjects on the Mediterranean diet, ranging from 4% to 10%. Thus, the Mediterranean diet demonstrated beneficial effects on liver imaging and histology features, with an inverse association between adherence to the Mediterranean diet and the severity of liver damage [43].

In the present study, we have observed both effects on MAFLD, specifically liver steatosis, and the reduction in PLC is associated with weight loss induced by bariatric surgery and the beneficial effect of the Mediterranean diet. In those patients with greater adherence to the Mediterranean diet, a significantly higher weight loss and significantly greater PLC reduction were observed. The efficacy of the Mediterranean diet in improving liver steatosis can be derived from the high antioxidant profile of this diet and its food components. Oxidative stress is related to liver steatosis and obesity, and a healthy diet can support the improvement of the inflammatory status related to both these conditions. The results of this study point to the importance of trying to maximize compliance with the prescribed Mediterranean diet. This may require closer monitoring by nutritionists to raise patients’ awareness of the importance of strict adherence to the diet, resolve any doubts, and reinforce appropriate behavior. This would not only affect the improvement of hepatic steatosis but would also be associated with greater weight loss, a higher rate of improvement of associated comorbidities, and probably less weight regain in the medium to long term.

Referring to the biochemical values, a significant improvement in the glycemic and lipid profile was observed. Furthermore, a significant reduction in AST and ALT was also shown after surgery. In addition, a greater improvement in fasting glucose, triglycerides, and liver enzymes was determined among those patients with a high adherence to the Mediterranean diet. Consequently, the Mediterranean diet seems to have a synergistic effect with bariatric surgery in the improvement of lipid and glycemic profiles but also in the reduction in plasmatic levels of liver enzymes, revealing a decrease in hepatolysis.

### Limitations

The study’s main limitation is the small sample size, but this is just a pilot study, whose results must be confirmed in further studies with larger sample sizes. Consequently, we cannot conclude from the present study if the greater reduction in liver steatosis was secondary to the greater weight loss or an additional effect of a higher adherence to the Mediterranean diet. A multivariate regression analysis would not yield statistically significant results to adjust for potential confounding factors and strengthen the conclusions of the study. The patients included in the study presented a BMI below 45 Kg/m^2^, to ensure that they could fit inside the MRI machine. This implies that the results obtained cannot be extrapolated to all morbidly obese patients. 

Despite biochemical parameters showing a postoperative significant improvement of the lipid and glycemic profile, we must take into consideration that the preoperative values from the patients diagnosed with dyslipidemia or type 2 diabetes were obtained under pharmacological treatment, while postoperative determinations were without treatment. This implies that the impact of the improvement is probably higher than is reflected in the values. Furthermore, preoperative pharmacological treatment might have also some effects on liver enzyme values or even on the PLC. Future studies must take these issues into consideration in their study design to obtain the most accurate biochemical and PLC results.

In addition, an assessment of body composition parameters, especially the percentage of body fat, determined by bioimpedance, would have been interesting. Unfortunately, these data were not available. Future studies must also consider including this assessment.

Referring to the Mediterranean diet, as previously mentioned, the prescribed diet followed a balanced Mediterranean-style pattern (its percentage composition consisted of 51% carbohydrates, 23% protein, and 26% fat). However, the specific calculation of grams of proteins per Kg of body weight was not performed. This would have been interesting to ensure a correct protein intake, aiming to reduce, in this way, the excess loss of lean mass associated with the loss of fat mass, in the context of postoperative weight loss after bariatric surgery. Additionally, although a common pattern was followed, this study did not include a specific analysis of energy intake, macronutrients, and micronutrients consumption. Future studies must also consider including this analysis. 

The assessment of adherence to the Mediterranean diet was performed with the KIDMED test. This tool is originally intended for children and adolescents, but we have used it in this project in an adult population, because our group was more used to managing with this test, as it is considered easier to understand than other tools. We have realized that many morbidly obese patients present a low cultural level, and we considered this test more appropriate for them. Anyway, we must accept as a limitation of the study that the adherence assessment was conducted with a tool not designed for an adult population. Future studies should analyze the accuracy of other tests. 

Moreover, it could have been interesting to characterize the diet used by the patients before bariatric surgery and to know their preoperative adherence to the Mediterranean diet. Probably those with the highest preoperative adherence are also likely to be those with the highest postoperative adherence. Unfortunately, these data were not recorded before surgery.

## 5. Conclusions

Liver steatosis significantly reduces after Roux-en-Y gastric bypass as a bariatric procedure. This reduction is further improved when associated with a high adherence to the Mediterranean diet.

## Figures and Tables

**Table 1:** Foods included in the diet.

Vegetables	Spinach, chard, aubergines, watercress, endive, lettuce, cauliflower, mushrooms, leeks, asparagus, endive, cabbage, cucumber, peppers, tomatoes, green beans, beetroot, carrot, artichoke, or Brussels sprouts
Cereals	Pasta, semolina, rice, tapioca, potato, bread, or unsweetened biscuits
Legumes	Lentils, chickpeas, or beans
Fruits	Apple, pear, orange, peach, kiwi, peach, melon, watermelon, or strawberry
Protein sources	White fish, chicken, turkey, rabbit, beef, or eggs
Dairy products	Skimmed milk, skimmed yogurts, or fresh cheese
Fat source	Olive oil

**Table 2:** Individual issues assessed in the KIDMED test.

	Moderate Adherence	High Adherence
1. Have a fruit or fruit juice every day.	100%	100%
2. You eat a second fruit every day.	75%	87.5%
3. You eat fresh, raw, salad, or cooked vegetables regularly once a day.	75%	87.5%
4. You eat fresh fish regularly (at least 2 or 3 times a week).	50%	75%
5. You go once or more per week to a fast-food center (e.g., hamburger restaurant).	16.7%	0%
6. Likes pulses and eats them more than once a week.	33.3%	50%
7. Eats pasta or rice almost every day (5 days or more per week).	25%	25%
8. Eats a cereal or cereal derivative (bread, toast, etc.) for breakfast.	100%	100%
9. Eat nuts regularly (at least 2 or 3 times a week).	16.7%	37.5%
10. Use olive oil at home.	100%	100%
11. Do not eat breakfast.	0%	0%
12. Have dairy for breakfast (milk or yogurt, etc.).	25%	50%
13. Eats industrial pastries for breakfast.	8.3%	0%
14. Eat 2 yogurts and/or 40 g of cheese every day.	25%	50%
15. Eat sweets and candies every day.	0%	0%

**Table 3:** The distribution of pre- and postoperative anthropometric measurements according to the adherence to the Mediterranean diet.

	Moderate Adherence (n = 12)	High Adherence (n = 8)	*p*
Preoperative weight	107.9 ± 17.9	106.5 ± 18.3	NS
Preoperative BMI	41.7 ± 3.5	41.1 ± 3.3	NS
Postoperative weight	78.1 ± 8.6	70.7 ± 7.4	0.048
Postoperative BMI	30.2 ± 2.7	27.3 ± 2.5	0.041
Percentage of Excess weight loss	68.9 ± 5.0	85.7 ± 5.4	0.027

**Table 4:** The distribution of pre- and postoperative PLC according to adherence to the Mediterranean diet. *p* represents the differences between moderate adherence and high adherence to the Mediterranean diet. D expresses the differences between pre- and postoperative values.

	Moderate Adherence (n = 12)	High Adherence (n = 8)	*p*
Preoperative PLC (%)	14.5 ± 9.5	13.8 ± 9.3	NS
Postoperative PLC (%)	4.5 ± 1.8	3.3 ± 1.6	0.045
D	<0.001	<0.001	

**Table 5:** Pre- and postoperative biochemical parameters.

	Preoperative	Postoperative	*p*
AST (U/L)	26.3 ± 18.1	17.7 ± 9.2	0.025
ALT (U/L)	36.9 ± 19.6	19.1 ± 10.6	0.019
Total cholesterol (mg/dL)	221.2 ± 39.5	205.2 ± 22.7	0.16
Triglyceride (mg/dL)	178.2 ± 31.5	95.3 ± 16.3	0.001
HDL-cholesterol (mg/dL)	46.5 ± 12.3	61.4 ± 13.8	0.001
LDL-cholesterol (mg/dL)	128.7 ± 23.4	117 ± 21.1	0.216
Fasting glucose (mg/dL)	106.5 ± 34.2	84.9 ± 18.7	0.001
Glycated hemoglobin (%)	6.5 ± 1.7	5.1 ± 0.6	0.011

**Table 6:** Distribution of postoperative biochemical variables according to the adherence to the Mediterranean diet.

	Moderate Adherence	High Adherence	*p*
AST (U/L)	19.2 ± 9.5	16.3 ± 8.9	0.048
ALT (U/L)	21.0 ± 10.8	17.1 ± 10.4	0.042
Total cholesterol (mg/dL)	206.0 ± 22.7	204.6 ± 22.5	0.341
Triglyceride (mg/dL)	99.4 ± 16.4	91.3 ± 16.0	0.037
HDL-cholesterol (mg/dL)	60.2 ± 13.7	62.8 ± 13.9	0.089
LDL-cholesterol (mg/dL)	118.1 ± 21.1	116.1 ± 21.0	0.395
Fasting glucose (mg/dL)	87.8 ± 18.8	81.9 ± 18.5	0.044
Glycated hemoglobin (%)	5.2 ± 0.7	5.1 ± 0.6	0.412

## Data Availability

Data are unavailable due to privacy or ethical restrictions.
